# Bioengineering, augmented reality, and robotic surgery in vascular surgery: A literature review

**DOI:** 10.3389/fsurg.2022.966118

**Published:** 2022-08-19

**Authors:** Sara Condino, Roberta Piazza, Marina Carbone, Jonathan Bath, Nicola Troisi, Mauro Ferrari, Raffaella Berchiolli

**Affiliations:** ^1^ Department of Information Engineering, University of Pisa, Pisa, Italy; ^2^ EndoCAS Center, Department of Translational Research and New Technologies in Medicine and Surgery, University of Pisa, Pisa, Italy; ^3^ Division of Vascular Surgery, University of Missouri, Columbia, MO, United States; ^4^ Vascular Surgery Unit, Department of Translational Research and New Technologies in Medicine and Surgery, University of Pisa, Pisa, Italy

**Keywords:** biomedical engineering, vascular surgery, endovascular surgery, augmented reality, robotic surgery

## Abstract

Biomedical engineering integrates a variety of applied sciences with life sciences to improve human health and reduce the invasiveness of surgical procedures. Technological advances, achieved through biomedical engineering, have contributed to significant improvements in the field of vascular and endovascular surgery. This paper aims to review the most cutting-edge technologies of the last decade involving the use of augmented reality devices and robotic systems in vascular surgery, highlighting benefits and limitations. Accordingly, two distinct literature surveys were conducted through the PubMed database: the first review provides a comprehensive assessment of augmented reality technologies, including the different techniques available for the visualization of virtual content (11 papers revised); the second review collects studies with bioengineering content that highlight the research trend in robotic vascular surgery, excluding works focused only on the clinical use of commercially available robotic systems (15 papers revised). Technological flow is constant and further advances in imaging techniques and hardware components will inevitably bring new tools for a clinical translation of innovative therapeutic strategies in vascular surgery.

## 1. Introduction: Biomedical engineering in vascular surgery

One of the definitions of biomedical engineering is *“the application of engineering principles, practices, and technologies to the fields of medicine and biology especially in solving problems and improving care (as in the design of medical devices and diagnostic equipment or the creation of biomaterials and pharmaceuticals)”* (1). This concept raises growing interest and approval thanks to the proliferation of medical implants, such as pacemakers and artificial hips, to more futuristic technologies such as stem cell engineering and 3D printing of biological organs, with the aim of improving life quality and medical healthcare at all levels, from diagnosis to treatment assessment and subsequent recovery.

Vascular surgery is one of the medical research fields in which technological advances, achieved through biomedical engineering, have contributed to significant improvements in open and endovascular surgical techniques in all arterial districts.

The first technological breakthrough can be considered to be the discovery of X-rays in 1895, which became relevant in the vascular field only a few decades later when a tolerable contrast agent for living humans was discovered, and it was possible to perform the first arteriography in a human being by direct puncture of the carotid artery (2). Thereafter, conventional angiographic methods have been constantly refined to improve the procedure’s safety and diagnostic efficiency.

The unceasing development of new surgical equipment and techniques has provided surgeons with the ability to perform more complicated procedures and successfully treat more challenging lesions in elderly and sicker patients. The innovation of endovascular aneurysm repair for patients with abdominal aortic aneurysms was a milestone in the evolution of vascular surgery into the endovascular era (3).

Developments in vascular surgery are not limited to the technical part of the operative procedure. In fact, other disciplines such as radiology are also involved. Computed tomography (CT) scan, ultrasound-Doppler imaging, and magnetic resonance imaging (MRI) are a combination of physics and electrical engineering. Nowadays, imaging is a biomedical engineering discipline in its own right, integrating signal processing and computational techniques. The acquisition of trustworthy morphological and functional data on the target area is essential for deciding the feasibility of an intervention and for planning, guiding and performing a specific procedure as well (4).

Recently, augmented reality (AR) technology has been successfully helping surgeons during image-guided surgery (IGS), integrating surgical navigation with virtual planning simultaneously with the real patient anatomy (5).

The introduction of robotics represented another major step forward for different surgical specialties, facilitating and improving the performance of minimally invasive surgery. Robot-assisted surgery has been brought into the area of vascular surgery to enhance laparoscopic vascular and endovascular skills such as a relatively difficult manipulation of instruments and long suturing times for anastomoses and clamping of the aorta or pelvic arteries.

In biomedical engineering, a variety of disciplines, such as mechanical engineering, electrical engineering, chemical engineering, materials science, chemistry, mathematics, science, and computer engineering, is integrated with human biology to improve human health and reduce the invasiveness of surgical procedures. However, the efforts to explore in detail the impact of all these sub-disciplines and/or technologies could be ineffective and confusing. Considering that, this paper aims to review the most cutting-edge technologies of the last decade involving the use of augmented reality devices and robotic systems in (endo)vascular surgery.

## 2. Search protocol and selected studies

Based on the literature search carried out by the authors of this study, there are currently no reviews in the literature focused on the use of AR in vascular surgery, apart from the work of Lareyre et al. (6) which, however, only analyses works involving the use of Head-Mounted Displays and Smart Glasses. Therefore, a review of the current literature was carried out to allow an all-around assessment of AR technologies, including the different technologies available for the visualization of virtual content. Whereas, with respect to robotic platforms introduced to assist both laparoscopic and endovascular vascular procedures, the literature of recent years is rich in clinical reviews (7–10) focusing on robotic applications in one or both of these surgical applications. For this reason, in this manuscript, only studies with bioengineering content that highlight the trend of research in robotic vascular surgery were considered, excluding works focused only on clinical use of these systems.

The PubMed database was used to identify studies, written in English, related to the use of AR and the employment of robotic technology for vascular surgery. The search period was from January 2010 to April 2022 inclusive.

To perform the initial review process, based on paper title search, two different combination of keywords were used as follows:

• (“Augmented Reality” AND “Vascular”) OR (“Augmented Reality” AND “Endovascular”);
• (“Robot” AND “Vascular Surgery”) OR (“Robot” AND “Endovascular Surgery”).

With the exception of the keywords combination, which is different for the two investigated topics, the records selection protocol was the same. Indeed, after the collection of papers and the exclusion of duplicates, records were screened to filter out reviews, editorials, and commentary which were not under consideration for this work. Then, the remaining records were screened through abstract reading to exclude out of topic publications.

As concerns the use of AR technology in the field of vascular surgery, a total of 86 records were identified through the studies collection from online digital library, and after removing 7 duplicates, 2 commentaries, and 6 reviews, the remaining abstracts’ records were examined to exclude publications related to other surgical specialties (e.g., cerebrovascular surgical procedures, duodenopancreatectomies, etc.). The final number of publications considered relevant for the review was 11. Whereas, regarding the involvement of robotic systems in the field of vascular surgery, 63 records were identified through PubMed database searching, and after removing 5 duplicates, 1 editorial, and 14 reviews, the remaining records were screened through abstract reading to exclude out of topic publications. Eventually, 15 articles were considered relevant for the present review.

**Figure 1:**
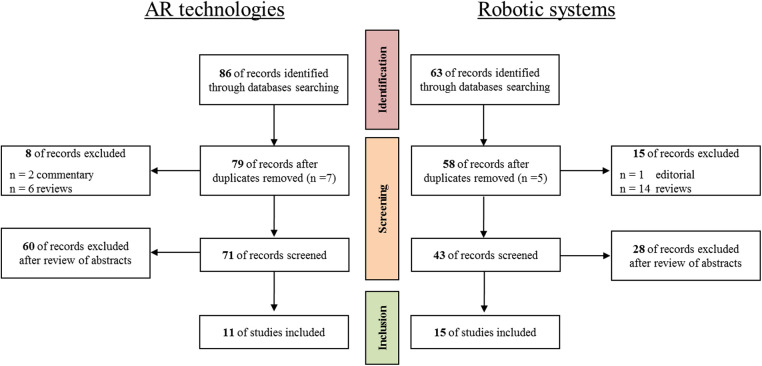
The flow chart for the selection of studies regarding the AR technologies (on the left) and the robotic systems (on the right) proposed in both vascular and endovascular surgery.

The flow chart for the selection of studies is shown Figure 1.

## 3. AR in vascular and endovascular surgery

In the following sections, the current diffusion of AR technologies in the field of vascular surgery was analyzed, examining for which specific applications these technologies have been used/proposed in both open and endovascular surgery. Furthermore, the type of AR displays adopted, the expected benefits, and the limitations of the current technology are reported, with an assessment of the maturity status of AR solutions in the vascular field. The revised records of this section are listed and described in Tables 1.

**Table 1:** AR technologies in vascular and endovascular surgery.

Author	Surgery type	Application	AR modality	Setup
Gao (11)	Endovascular	Catheter navigation	HMD	Vascular phantom
García-Vázquez (12)	Endovascular	Tools navigation in EVAR	HMD	Torso phnatom
Lu (13)	Endovascular	Retrograde peroneal access	AR glasses	Clinical
Rynio (14)	Endovascular	Planning and navigation in EVAR	HMD	Clinical
West (15)	Endovascular	Navigation and stentgraft deployment	HMD	Animal model
Mialhe (16)	Endovascular	Peripheral and carotid angioplasty, EVAR	HMD	Clinical
Mialhe (17)	Endovascular	Lower limb angioplasty	HMD	Clinical
Parrini (18)	Endovascular	Tool navigation	HMD	Vascular phantom
Cheng (19)	Endovascular	Tools navigation	2D display	*Not specified*
Aly (20)	*Not specified*	Groin incision guidance	Smartphone	Clinical/vascular phantom
Jeon (21)	*Not specified*	US-guided vascular acces	Microprojector	Vascular phantom

### 3.1. Visualization modalities

Available display technologies to provide the user with AR visualization include 2D monitors, hand-held displays (e.g., mobile phones and tablets), head-mounted displays (HMDs), and spatial projection-based AR displays.

Eight papers out of 11, report the use of HMDs or Smart Glasses (11–18). These displays provide the user with an egocentric viewpoint and allow operators to work hands-free, and according to recent literature (22, 23), they have been deemed the most ergonomic solution for applications including manual tasks performed by the user under direct vision, like what happens in open surgery. However, as is evident in this study, there is growing interest in the literature on the use of this type of display even for minimally invasive procedures, such as endovascular procedures.

Endovascular surgeons are traditionally forced to turn their heads away from the surgical field to view the standard fluoroscopic monitor; the use of a wearable display instead can provide surgeons with an alternative screen in front of their eyes, allowing them to keep their attention focused on the operative field. Evaluation of the potential benefits of wearable displays for performing fluoroscopically guided interventional procedures versus traditional monitor visualization is currently being explored in several surgical fields (e.g., fluoroscopically guided minimally invasive spinal instrumentation surgery (24), including endovascular surgery (16, 17). The assumption of these studies is that wearable displays have the potential to facilitate better concentration on surgical tasks by enhancing ergonomic efficiency during surgery.

Most of the studies selected in this review employ Microsoft HoloLens as HMD (12, 16, 17, 14, 15). Microsoft HoloLens is designed with an optical see-through (OST) approach: virtual reality (VR) data are projected on a semitransparent display in front of the user’s eyes and the natural view of the real world is preserved, allowing the natural synchronization of visual and proprioceptive information, and complete situational awareness. This approach fits well in the surgical domain as it offers an instantaneous full-resolution view of the real world, however, in OST displays the spatial coherence between the VR content and the real scene is still suboptimal, and perceptual and technological issues still limit their employment when a high virtual to real spatial alignment (i.e., registration) is required for accurately guiding manual tasks in the peripersonal space (22). Clearly, these issues do not arise when the HMD is used solely as an alternative monitor for viewing preoperative information (e.g., 3D anatomical models extracted from preoperative computed tomography angiography (CTA) or intraoperative information (e.g., fluoroscopic images) in applications that do not require image-to-patient registration. On the contrary, video see-through (VST) systems, like the one employed by Parrini et al. (18), can offer an accurate registration of virtual content to the real scene at the cost of a camera-mediated view. In this proposed application (18), for example, the HMD is used to visualize the vessel centerlines superimposed on the patient body to guide endovascular tools.

Three papers out of 11, describe AR applications based on displays other than HMDs. More particularly: a traditional 2D display is used to display the AR scene in the application proposed by Cheng et al. (19); Aly et al. propose a low-cost handheld solution based on smartphone hardware (20); Jeon et al. employ a microprojector, attached to an ultrasound (US) probe, to project the US image over the patient skin for simplifying ultrasound-guided vascular access (21).

### 3.2. Surgical applications

Most of the analyzed records (9 papers out of 11) focus on the use of AR in endovascular procedures. Among them, some papers (11, 12, 18, 19) do not focus on a specific surgical procedure but describe more extensively the usefulness of AR in endovascular surgery. The rest propose the following use cases: endovascular treatment of abdominal aortic aneurysms (EVAR) (12, 16, 14, 15), peripheral angioplasty (13, 17, 16), and carotid angioplasty (16). The remaining papers concern: (1) an application useful for a wide variety of vascular and endovascular procedures, intended to guide groin incisions (20), (2) a simplified AR device for ultrasound-guided vascular access (21).

Emerging applications of AR in surgery include: preoperative systems for surgical planning and patient-specific rehearsal, intraoperative systems for navigating complex procedures and/or easing the visualization of preoperative/perioperative patient data, and simulation systems for surgical training. According to the findings of this review, in the field of vascular surgery, AR functionalities have been mainly explored for developing innovative intraoperative platforms to assist the surgeon during the navigation of endovascular tools and/or to furnish an ergonomic tool for visualizing patient data during the intervention.

Cheng et al. present an AR framework, designed to be integrated with a robotic device to navigate endovascular tools, that can also be used for surgical training/planning in ultrasound-guided endovascular procedures (19). The novelty of the system lies in the capability of allowing the surgeon to pre-plan an optimal path (which can be adjusted during operation). Moreover, the system features mixed reality functionalities fusing real-world elements (US images) with virtual synthetic elements (3D model of the anatomy, virtual medial axis of blood vessels, planned navigation path) in a single 3D scene to enhance the surgeon’s visual perception. The proposed platform employs an electromagnetic (EM) tracking module to track in real the US probe and the endovascular tools (i.e., the catheter tip) via built-in EM sensors.

EM tracking is a widely used technique that enables real-time tracking of surgical tools without line-of-sight restrictions and without ionizing radiations (25). The use of EM sensors for the tracking of endovascular instrumentation has already been proposed in the literature for the development of endovascular navigation systems based on virtual reality (26–29), and as it emerges from this study is also being evaluated for the development of AR endovascular navigation systems (19, 11, 12, 15).

The system implemented by Gao et al. (11), similarly to what has been proposed by Cheng et al. (19), provides intraoperative assistance to the surgeon for catheter navigation through AR visualization of the vasculature virtual model, the optimal catheter trajectory, and the current position of the catheter that is tracked through an EM localization system. The VR content is spatially aligned to the patient’s anatomy (i.e., registered), and it is displayed via a HMD.

The navigator developed by García-Vázquez et al. (12) tackles the issues of radiation exposure and contrast agent administration during EVAR interventions by using a multidisciplinary approach to guide the endovascular tools: EM localization of endovascular tools, and AR visualization (via a HMD) of the endovascular tools’ position, the 3D models of the skin and vascular structures superimposed on the patient anatomy. Additionally, the authors envision the use of 3D ultrasound, streamed from the US system to the HMD, for guiding endovascular tools and updating navigation with intraoperative imaging.

Finally, among the systems featuring EM tracking technology, there is the platform proposed by West et al. (15) for assisting in the intraprocedural deployment of endovascular stent-grafts during complex EVAR procedures. This system allows the visualization of 3D models of the patient anatomy extracted from preoperative images and offers numerical feedback for controlling the endograft landing zone and the alignment with the aorta ostia, via EM tracking of the stent-graft.

Four out of 11 papers concern an application specifically designed for intraoperative navigation, without providing EM localization of the instrumentation (20, 13, 18, 21). More particularly, the system developed by Aly et al. (20) is conceived to assist the surgeon in the localization of vascular structures via the AR visualization of a 3D model of the patient vasculature, extracted from preoperative CTA, registered to the patient body and displayed via a smartphone. Lu et al. propose an application to ease retrograde peroneal access for the endovascular treatment of critical limb ischemia (13). The application is based on the use of an AR navigation system (Xiamen Minwei Limited Company, Xiamen, China) featuring AR glasses that are employed by the authors to visualize the recommended puncture path (site, depth, and angle of puncture) to the target vessel. Parrini et al. propose using a VST HMD to visualize vessel centerline extracted from volumetric radiological images (e.g., CT, MRI, or 3D US) registered to the patient body (18). The proposed innovative application is the assistance to freehand guidance of magnetic endovascular devices. Finally, Jeon et al. propose a simplified AR device for ultrasound-guided vascular access (21): US images are transmitted to a microprojector (attached to the US probe) and projected on the patient skin. The projected images are calibrated so that the acquired anatomical structures are displayed at full scale.

The remaining 3 records concern the evaluation of the intraoperative use of AR for enhancing the ergonomic efficiency of patient data visualization. More particularly, the intraoperative use of Microsoft HoloLens to develop “screenless display” endovascular interventions has been proposed. In (16) perioperative angiography images were broadcast live in the HoloLens, with no latency, and successfully visualized by the surgeon during three interventions: peripheral angioplasty, carotid angioplasty, and EVAR procedure. In (17), during a lower limb angioplasty, up to four images originating from different sources were displayed simultaneously including: 2D angiography, operative vital signs monitoring, 3D fusion image, and 3D CT scan reconstruction. Finally, Rynio et al. used the HoloLens device during an EVAR intervention to visualize 3D models of the aneurism with its thrombus and adjacent bones, and a 2D image containing the volume rendering reconstruction with arterial diameters and planning notes (14). Moreover, they explored the manual image fusion with fluoroscopic data: for this purpose, the hologram was placed in front of the angiographic monitor, and scaling/rotation procedures were performed to manually register the 2 modalities based on bones.

### 3.3. Perceived advantages and current limitations

As it emerges from literature, an increasing number of surgeons perceive the potential benefits of using AR in vascular and endovascular procedures, indeed this technology may facilitate visualization and navigation during surgical procedures and could improve the surgical workflow (14).

As for intraoperative visualization of patient data, AR systems offer the opportunity to easily display many forms of 2D and 3D medical data preserving the 3D spatial relationships between the anatomical structures. According to (14) who tested the Microsoft HoloLens in the surgical room, *“until now, our workflow was to print several images of the volume rendering from different angles. We found that data useful whenever a problem arose due to difficult anatomy (branch takeoff angles, tortuosity, etc). However, such data always were 2D, and we could not reach for views other than those already prepared. The AR approach is far most helpful, being available all the time and enabling rotation in all angles with preservation of structural relationships.”* In addition, to making the information content of radiological images more intuitive and easier to use, such visualization systems could reduce the frequency of operator head-turning, and thus the risk of inattention. Moreover, HMDs such as Microsoft HoloLens can be operated by voice commands and gestures, they do not need to be handled, leaving hands free and maintaining the sterility of the environment (16). In the field of navigation, AR has the potential to lower contrast material volume and radiation exposure without interfering with the operator’s routine activities (13, 15), to ease procedures and improve their accuracy (15), to decrease surgical time (15).

This review also highlights several limitations of current technologies that hinder their widespread use in clinical settings. For example, according to (14), some drawbacks of Microsoft HoloLens V1 include: non-negligible weight (579 g), which can cause some fatigue to the operator’s neck, especially in case of prolonged use; restricted binocular vision field (30° diagonal) (14); limited battery life (between 2 and 3 hours of working time) meaning it may not be sufficient for long procedures. The new version of Microsoft HoloLens, partly mitigates these issues, indeed HoloLens V2 features an improved field of view (52° diagonal) and offers more comfortable wearability; however, currently available HMDs are still far from the ideal AR display, which, according to Rolland et al., should be *“conceived as a transparent interface between the user and the environment, a personal and mobile window that fully integrates real and virtual information”* (30).

Another perceived technical issue, that more broadly afflicts AR systems independently of the selected display is the difficulties in obtaining and maintaining overlapping between the AR content and the patient anatomy (20, 18, 12). None of the analyzed records employ a non-rigid image-to-patient registration technique or incorporate a method for the intraoperative update of the vascular 3D models. Moreover, they mostly employ external artificial markers or anatomical landmarks on the body surface for registration and this intrinsically limits the registration accuracy.

The registration of intra-abdominal structures that move and deform with ventilation and heart-beat is proven to be a challenge for current computer-assisted systems. As pointed out by Aly et al. (20) registration deformation is not a major issue when the vascular structures targeted by the AR application are relatively fixed and the patient is not repositioned intraoperatively. However, in this case, the effects of soft tissue deformation due to the interaction with the surgical instrumentations may reduce the system accuracy. As suggested by García-Vázquez et al. a possible solution to this problem could be the use of intraoperative data (such as intraoperative US volumes) to acquire an updated model of the patient anatomy, combined with non-rigid registration techniques (12).

### 3.4. Maturity of the AR technology, level of testing and certification as a medical device

Most of the AR platforms described in the selected records are based on the use of a commercial display, not specifically conceived for medicine or surgery (e.g., Microsoft HoloLens), coupled with a prototype software architecture. An exception is the CarnaLife Holo, employed by (14), a module of the telemedicine system “CarnaLife,” which is certified as a medical device supporting diagnostics, class IIb. Moreover, no information has been retrieved on the internet on the “AR navigation system” (Xiamen Minwei Limited Company, Xiamen, China) used by Lu et al. (13).

Five records out of 11 (19, 11, 12, 21, 18) are feasibility studies in an in-vitro setup to qualitatively and/or quantitatively test some technical aspects of the proposed system. For example, the manuscript by García-Vázquez et al. reports a qualitative evaluation of the AR misalignment of a system based on Microsoft HoloLens and a rigid registration algorithm (12). Moreover, the same manuscript reports a quantitative evaluation in the visualization of US volumes. According to the authors, both the registration accuracy and the visualization latency should be improved before clinical applications. The latter is 259 ± 86 ms and should be at least 100 ms to display real-time 3D US volumes on HoloLens.

One record reports a feasibility study on a human volunteer to test in a qualitative way (via landmarks palpation) the registration accuracy of a system designed to assist in the intraoperative localization of vascular structures (20). Moreover, it also reports the results of an in-vitro study on a mannequin demonstrating the stability and the accuracy of the positional/rotational tracking reachable with a hybrid gyroscopic and optical tracking approach using low-cost smartphone hardware.

Four manuscripts (13, 17, 16, 14) are clinical case reports on a limited number of surgical patients (maximum 3 in (16)), showing the feasibility of using Microsoft HoloLens to develop “screenless display” endovascular interventions.

Finally, the manuscript by West et al. tests the “Three-Dimensional Holographic Guidance, Navigation, and Control (3D-GNC) prototype” for endograft positioning in porcine aorta, comparing the system performance with 2D X-Ray fluoroscopy (15). Technical success for the use of 3D-GNC (without fluoroscopy or contrast-dye administration) to orient and position the endovascular device at each renal-visceral branch ostium was 100%, and according to obtained results, the proposed system is able to reduce procedure time (by 56%) and to improve overall orientation accuracy (by 41.5%). Positioning accuracy was comparable for both techniques.

It may be concluded that in the case of systems designed for intraoperative navigation, although in some cases the results obtained in-vitro or on animals are very promising, to date there are no clinical studies and the devices are not certified for surgical use. The use of AR technologies for intraoperative visualization of patient data is certainly a more mature application, but to date, the number of clinical studies is still limited to drawing definitive conclusions on the fascinating potential of AR.

## 4. Robotic surgery in vascular and endovascular surgery

Robotic technologies are increasingly being adopted in a variety of surgical disciplines to facilitate and improve the performance of minimally invasive surgery. In the following paragraphs, the main findings of existing reviews in the field of robot-assisted laparoscopic vascular surgery and endovascular surgery are first summarised. Finally, the leading bioengineering topics of current studies are reported in order to analyze the latest scientific trends in robotic vascular surgery.

### 4.1. Robot-assisted laparoscopic vascular surgery

The first robot-assisted vascular surgery dates back to 2002, when Wisselink et al. reported the first two cases of robotic technology being used for laparoscopic aortobifemoral bypass grafting (31). During the surgery, three robotic positioner arms were connected to the operating table rails: one for a 30-degree endoscope (Aesop Endoscope Positioner, Computer Motion) on the right, and two for surgical instruments, consisting of a needle driver and a grasper (Micro Joint Heavy Needle Driver, Micro Joint De Bakey Grasper) on the left side of the patient. The aim of using the robotic technology was to simplify endoscopic manipulation by increasing the degrees of motion and facilitating hand-eye coordination.

Since then, two advanced robotic surgical systems have been mainly used in laparoscopic vascular surgery: the da Vinci (Intuitive Surgical Inc, Mountain View, Calif) and Zeus (Computer Motion Inc, Santa Barbara, Calif) systems, which are Leader-Follower robots with similar capabilities (10). The production of the Zeus robot has been discontinued, while in the last decade, the da Vinci robotic surgical telemanipulator has been used for several vascular procedures, that, according to a recent literature review (8), include: robotically assisted repair of abdominal aortic aneurysms (AAA), thromboendarterectomy of the aorta and pelvic artery, iliofemoral and aortofemoral bypass, thoracofemoral bypass from descending thoracic aorta, splenic artery aneurysm, renal artery reconstruction, robotic treatment of type II endoleak after endovascular aneurysm repair, robotic surgery for celiac artery compression syndrome, and robotic-assisted central venous reconstruction.

Robot-assisted laparoscopic surgery offers several advantages over traditional open surgery, including less intraoperative bleeding, early restoration of gastrointestinal activity, rapid postoperative recovery, good surgical wound healing, excellent cosmetic results and healing of surgical wounds, and dramatic reduction of the occurrence of incisional hernia. In addition, the benefits of robotic technologies in terms of improved dexterity, restoration of proper hand-eye coordination and better visualisation can further enhance the surgical outcome. For example, according to (8), surgical robots can allow vascular anastomoses to be performed more quickly and easily than classic laparoscopic surgery, eliminating the difficulties of handling laparoscopic instruments. Similarly, in the field of micro- and supermicrosurgery, robotic systems are proving to successfully bridge limitations related to the precision and dexterity of the surgeon’s hands, paving new options for micro-reconstruction procedures as well. Most recently, the Symani (Medical Microinstruments - MMI, Calci, Italy) robot, a teleoperated microsurgical system, has been used for the first time on humans to perform ten microanastomoses (i.e. lympho-venous and arterial anastomosis for lymphatic reconstruction) (32).

However, to date, data from the existing literature are limited to drawing a conclusion regarding the efficacy of robotic technology in vascular surgery (10), given that most studies are mainly limited to individual cases (Stadler’s group, which reported on a series of 285 procedures (33), is an exception). At present, robot-assisted vascular surgery has not achieved great popularity and its use is still limited to a few centers worldwide. According to (10), one possible explanation is that the da Vinci system is not approved for this medical field, moreover, robotic surgery is generally more expensive than conventional procedures and has been associated with longer operating times for many types of procedures. However, with regard to the latter aspect, as pointed out by Soomro et al., most of the existing comparative studies may have been conducted by surgeons who were still learning the robotic technology, and therefore the results may not reflect the potential benefits of this technology (34).

### 4.2. Robot-assisted endovascular surgery

Research on endovascular interventional robots has been carried out since the end of the twentieth century. According to the difference in catheter actuation, vascular robots can be classified into four categories: magnetic, pull-wire (tendon drives), smart material-actuated, and hydraulically driven (35).

Among these, the most studied systems, which have resulted in commercial solutions, are magnetic and pull-wire systems. Magnetic systems are based on the use of catheters incorporating small magnetic implants in their tip, which act as magnetic dipoles, and two or more guide magnets, placed close to the surgical table, are used to generate a magnetic field that can be controlled to deflect the catheter tip to the desired position. Pull-wire actuation is another well-studied approach for developing steerable catheters and to simplify their control in the arterial tree.

Regardless of the technology used, robotic systems allow better control of the distal tip of the catheters, so they can allow better access to difficult anatomies, as well as better catheter stability. Finally, they allow teleoperation functionalities: the patient can be treated remotely, resulting in lower exposure to X-ray irradiation for the physician (35).

Commercial robotic platforms include the Magellan and Sensei systems (both owned by Auris Health, Inc), the CorPath system (Corindus Vascular Robotics, Inc), and the Amigo (Catheter Precision, Inc). In addition, another commercial platform is the Niobe system (Stereotaxis, Inc) which is based on a magnetically guided mechanism.

The Sensei robotic catheter was approved in 2007 by the FDA for use in cardiac mapping and ablative procedures (9), followed by the Magellan system, the first purely vascular robot that received FDA approval in 2012 (10). The Sensei system is equipped with pull-wire steerable sheaths, allowing remote manipulation of catheters via a three-degree-of-freedom (3-DOF) joystick, and has been employed successfully in a range of interventions, including cardiac ablation and standard and complex endovascular aneurysm repairs (9). Furthermore, the literature reports a clinical study on the use of this technology for iliac artery and superficial femoral artery cannulation (10).

The Magellan system, specifically designed for peripheral vascular intervention, consists of a remote workstation and a robotic arm that delivers the steerable catheter. The workstation includes a controller, a 7-DOF joystick, and a control screen. The system allows the operator to remotely control the catheter insertion/withdrawal, multidirectional movement, angulation, rotation, and torque position (9). According to (10) use-cases reported in the literature include: visceral and renal vessel cannulation during FEVAR/BEVAR, catheter placement in aortic arch during TEVAR, EVAR gate cannulation, and carotid artery angioplasty.

The CorPath system, which was designed for procedures in the whole cardiovascular system, is equipped with a robotic cassette compatible with commercial devices, allowing control of all three interventional devices, i.e. guidewire, catheter and balloon/stent catheters. As reported by Cruddas et al., the use of this robotic platform for the treatment of symptomatic peripheral arterial disease has been associated with improved technical success, shorter procedural times, and reduced use of fluoroscopy and contrast (9). According to (10), use cases reported in the literature also include percutaneous angioplasty of the superficial femoral artery and carotid angioplasty. However, large studies and cost-benefit analyses on its use in daily practice are lacking in the literature (9).

The Amigo system, designed for radiofrequency ablation and cardiac mapping, allows remote 3-DOF manipulation (insertion/withdrawal, tip deflection, and rotation) of standard steerable electrophysiological catheters.

Finally, the Niobe platform is a magnetically controlled robotic system, which requires a dedicated room and magnetically compatible equipment. The system can automatically control the orientation (3-DOF) of the distal tip of the catheter. The latter is softer than the tip of cable-operated catheters, thus reducing the risk of damaging vessel walls. According to (9) use-cases reported in the literature include coronary and peripheral arterial interventions.

As concluded in (9), none of the robotic systems mentioned have been used on a large scale, nor are they employed in clinical routine, so there is limited data available about their safety, effectiveness and efficiency. The main limitations of current systems include technical complexity, high cost, and, in most cases, difficulty of use with existing endovascular devices. These barriers need to be overcome to allow for a wider diffusion of robotic technology. From a technical point of view, it is essential to ensure the compatibility of robotic systems with a wide range of off-the-shelf equipment and their easy interchangeability, and an improvement in navigation technologies.

### 4.3. Bioengineering research trends in robotic vascular surgery

Based on the survey conducted in this study, the main research topics addressed in the last decade in vascular robotics include: the development and testing of advanced robotic functionalities to improve robot performance and human/robot interaction, the development of simulators to be used as a training system, and the study of the learning curve of surgeons during robotic surgery.

As for the first point, research was performed to:

1. Actively monitor the safety of robotic procedures and provide force feedback to the surgeon via haptic devices (36, 37).
2. Improve the user interface (37) and provide high-fidelity human-machine interaction (e.g, in (38), a novel master controller to obtain real-time detection of surgeon’s operation without interference to the surgeon is proposed. A real catheter is used as the operating handle, thus the surgeon’s feeling is similar to that in conventional endovascular interventions).
3. Design robotic systems not requiring the use of special proprietary tools, or modified catheters, to allow for wider use of the robotic technology and reduce its cost (36, 37).
4. Miniaturize endovascular devices and study magnetic propulsion methods (39).
5. Investigate the use of ultrasound imaging to navigate the endovascular tools to allow for a reduction of X-ray irradiation to the patient and contrast medium injection (39, 19).
6. Enrich the robotic platform with a framework for preoperative planning and simulation, plus advanced navigation functionalities integrating AR for improving the ergonomy of preoperative and intraoperative information visualization (19).
7. Optimise the gripping and manipulation performance of vascular interventional robots (40), modeling the hysteresis (i.e., the discrepancy between the input signals received at the proximal end of an endovascular tool and the movement that reflects at the tool’s distal part) in robotic catheterization (41).
8. Enhance the autonomy of the robot to expand human capacity and capability in human-robot collaborative surgery. Zhao et al. propose a model of human-robot collaborative surgery, where the robot is controlled by both the surgeon and a trained network (38). This would, for example, allow the robot to perform repetitive, low-risk surgical tasks autonomously under the supervision of a surgeon, while the surgeon can concentrate on complex, high-risk tasks and focus the cognitive load where it is really needed.
9. Evaluate the workflow and define the telecommunication requirements for telerobotic vascular interventions. This is paramount to allow for remote interventions to distribute advanced care to hospitals where no endovascular experts are available and to provide the possibility of remote proctoring (42, 43).

An important line of research concerns the development of effective simulation systems that provide surgeons with the opportunity to train in the use of new robotic technologies within a safe and repeatable environment, while reducing risks to the patient (44–46). Research in this field aims to achieve reliable simulation of anatomical deformations and high performance in real time (46), and furnish a comprehensive training environment that combines visual cues and force sensation to assist the novice for safe procedures execution reducing collision trauma (44, 45).

Finally, some literature studies (47, 48) were addressed to the evaluation of the learning curve during robot-assisted vascular procedures (in particular with the da Vinci robotic platform). The aim is to verify the complexity of the procedures, to study the speed of the learning process of robotic surgery techniques, and to verify if and how the traditional surgical experience influences the learning curve in robotic surgery. The results of these studies can provide important guidelines about the optimal modalities for the acquisition of the necessary skills for a profitable use of the robotic technology.

## 5. Discussion

More and more different technologies are being developed, replaced or integrated into standard clinical practice that inevitably affect the way care is received. The purpose of this literature review was to outline the basic concepts of augmented reality and robotic technologies with regard to vascular and endovascular surgery.

This work indicates that the potential benefits of augmented reality technology extend to both surgeons and patients. These can include reducing risk to the patient and operative time, as well as optimizing contrast and radiation exposure during radiological procedures, thanks to the development of intraoperative systems for surgical planning and navigation. The use of wearable or projection-based displays would allow surgeons to maintain the concentration on the operative field, avoiding repeated movements of the head toward standard fluoroscopic monitors, and providing an ergonomic tool for patient data visualization under the user’s direct vision. However, limitations such as the non-negligible weight and the restricted field of view of wearable displays, or the lack of non-rigid image-to-patient registration algorithms have to be investigated and solved to enable the AR usability in the future and to include it regularly in both clinical practice and training in vascular surgery.

The use of robotics in vascular surgery is well documented in the literature, despite none of the systems described above having been employed on a widespread basis. Aided by a robotic system, the surgeon’s movements are down-scaled into fine gestures, physiological tremor is eliminated, and the visualization is improved, thus simplifying those actions unachievable in traditional surgery. Moreover, a precise and teleoperated control of endovascular instruments allows an easy access to even the most complex anatomies and a reduction of X-ray exposure for the surgeon. Further refinements are needed to fully integrate this promising technology into the clinical environment, including advances in haptic feedback and compatibility with existing devices. Unfortunately, the high costs of robotic systems, along with those related to maintenance, and the lack of approval for use in the vascular field have contributed to their low popularity. However, concerns of addressing higher costs in favor of substantial health benefits for medical staff and patients should be considered.

The burgeoning field of biomedical engineering is the source of the most challenging technological advances in the healthcare field, affecting multiple aspects of the medical life of every patient, from the way the human body is examined for clinical conditions to the way major surgeries are ultimately performed. For this reason, there must be synergy between the different disciplines: engineers need to be aware of the many factors involved in the care and treatment process, just as clinicians need to be familiar with the engineering fundamentals behind the instrumentation they use in clinical practice and lend support in the research and development phases of new technologies. In this way, the integration of expertise derived from the medical and engineering worlds can offer patients increasingly precise, innovative, and more personalized care.
